# Systematic review and meta-analysis of positive long-term effects after intra-articular administration of orthobiologic therapeutics in horses with naturally occurring osteoarthritis

**DOI:** 10.3389/fvets.2023.1125695

**Published:** 2023-02-23

**Authors:** Anna Mayet, Yury Zablotski, Susanne Pauline Roth, Walter Brehm, Antonia Troillet

**Affiliations:** ^1^Department for Horses, Faculty of Veterinary Medicine, Leipzig University, Leipzig, Germany; ^2^Center for Clinical Veterinary Medicine, Faculty of Veterinary Medicine, Ludwig-Maximilians-University, Munich, Germany

**Keywords:** horse, degenerative joint disease, regenerative medicine, orthobiologics, autologous blood products, mesenchymal stromal cells (MSC), review–systematic, meta-analysis

## Abstract

Equine veterinarians face challenges in treating horses with osteoarthritic joint pain in routine veterinary practice. All common treatment options aim to reduce the clinical consequences of osteoarthritis (OA) characterized by persistent synovitis and progressive degradation of articular cartilage. A range of joint-associated cell types and extracellular matrices are involved in the not yet entirely understood chronic inflammatory process. Regeneration of articular tissues to re-establish joint hemostasis is the future perspective when fundamental healing of OA is the long-term goal. The use of intra-articular applied biologic therapeutics derived from blood or mesenchymal stroma cell (MSC) sources is nowadays a well-accepted treatment option. Although this group of therapeutics is not totally consistent due to the lack of clear definitions and compositions, they all share a potential regenerative effect on articular tissues as described in *in vivo* and *in vitro* studies. However, the current stage of science in regenerative medicine needs to be supported by clinical reports as in fact, *in vitro* studies as well as studies using induced OA models still represent a fragment of the complex pathomechanism of naturally occurring OA. This systemic review aims to determine the long-term effect of orthobiologic therapeutics in horses suffering naturally occurring OA. Thereby, a meta-analysis of randomized controlled trials (RCTs) is conducted to describe the efficiency and safety of intra-articular applied orthobiologics in terms of lameness reduction in the long-term. Using the PRISMA (Preferred Reporting Items for Systematic Reviews and Meta-Analysis) guidelines, thirteen studies met the inclusion criteria for the systemic review. Four of those studies have further been evaluated by the meta-analysis comparing the long-term effect in lameness reduction. Each study was examined for risk of bias. For data evaluation, a random-effects model was used, describing the overall outcome in a forest plot. The I^2^ statistic was used to assess heterogeneity. Results indicate, that orthobiologic therapies represent an effective long-term and safe OA treatment option. Due to the inhomogeneity of included studies, no statements are provided addressing specific orthobiologic therapies, affected joints, OA stage and horse's intended use. Future clinical trials should follow standardized study designs to provide comparable data.

## 1. Introduction

Osteoarthritis (OA) is an intensively researched condition in human and equine patients characterized by persistent articular inflammation leading to chronic synovitis, progressive destruction of articular cartilage, and consequently to a permanent loss of function and joint pain (1–3). Causative and stimulating factors of OA are still not fully investigated. In horses, the etiology of OA is assumed to be mainly post-traumatic. Therefore, OA in horses can be understood as the result of a failed repair of damaged articular and periarticular tissues. However, not only the nature of the initial structural tissue damage (repetitive microtrauma vs. single severe trauma), but also the degree and course of imbalance of the joint homeostasis seem to determine OA manifestation and progression (4).

In equine patients, joint related diseases including OA are considered the most common cause of lameness, as being involved in approximately 60% of all lameness cases (5–7). More than 70% of racehorses population suffer from lameness due to articular inflammation during their career (8, 9). However, the occurrence of OA is not only linked to high-speed and high-performance sport horse disciplines, such as horse racing (10–12) and show jumping (13, 14), but also to the increasing age of the patients (15–17). In OA-affected horses, the prognosis for long-term return to exercise and work on intended use varies between 30 and 50% and depends on the disease stage, the affected joints, and the horse's work level (17, 18).

In daily clinical practice, equine veterinarians face the challenge of treating OA as a persistent and chronic disease potentially affecting all joint associated tissues (10, 19, 20). Often the subsequent treatment choice is based on the veterinarian's personal experience, the owner's economic feasibility and the intended use for the horse in relation to disease stage. Although a broad spectrum of varying therapeutic concepts is stated (21, 22), conventional treatment options are limited in terms of modifying or reversing disease progression, thereby potentially being inferior in the long-term treatment success. However, the development of successful long-term treatment options is difficult, due to the intricate pathomechanisms of OA initiation as well as progression and the involvement of various cell types and extra-cellular matrices.

Recent studies have shown that biologic therapeutics derived from blood and mesenchymal stromal cell (MSC) sources hold a potentially regenerative potential for articular tissues *in vitro* (23–26) and *in vivo* (27–29). Beneficial clinical effects described after an intra-articular administration of biological therapeutics include reduction of lameness and joint effusion (30–32). It is assumed that clinically relevant effects of intra-articular administered blood products and MSCs in OA-affected joints in part are attributed to locally effective growth factors, cytokines, as well as secretomes and exosomes from delivered cells, which further innate on-site cell regeneration (33–35). Although the group of these so named orthobiologics or orthobiologic therapeutic agents is not totally consistent due to differences in manufacturing, processing and application, they all share potential regenerative effects on the described articular tissues proven *in vitro* (26, 36, 37) and *in vivo* (38–40) studies.

After more than 20 years of clinical experience in equine medicine, the use of intra-articularly applied orthobiologic therapeutics is considered as a safe and recognized treatment option for osteoarthritic joints today (41, 42). Yet, existing studies, which form the basis of our knowledge about the efficacy of orthobiologic therapeutics in equine medicine, differ in fundamental study design parameters like the availability of placebo groups or the type of researched OA (naturally occurring vs. experimentally induced OA). Consequences drawn from these studies are at best implemented in the treatment of clinical cases and provide evidence-based treatment concepts for equine OA. However, due to the heterogenicity of therapeutic products (blood-derived, tissue-derived), processing methods and components used (cell-free, blood-derived cells, tissue-derived cells), and treatment regimens (single injection, multiple injections), an unacceptably high number of subjects would be required to draw definitive conclusions. Therefore, the application of quantitative statistical methods summarizing primary data from clinical and experimental trials *via* meta-analysis is a useful tool to draw conclusions from a cohort of studies. The aim of the present study is to conduct a systematic review of current literature in the field of the intra-articular application of orthobiologic therapeutics in naturally occurring equine OA. Furthermore, a meta-analysis of *in vivo* and controlled studies has been carried out to assess the long-term effect of orthobiologic therapeutics on naturally OA-affected joints in horses.

## 2. Materials and methods

### 2.1. Definition of orthobiologic therapies

The present systematic review focuses on the following two intra-articularly applicable orthobiologic therapeutic concepts for equine OA.

#### 2.1.1. Mesenchymal stromal cells

Caplan described the first approaches of stem cell therapy in 1991, proposing potential differentiation into desired tissues (43). The characteristic differentiation potential of these cells has laid the foundation to prove therapeutic concepts in various fields of medicine where tissue regeneration and restoration are the aimed effects (44–49). In the process of clinical stem cell application, orthopedic diseases such as OA were becoming an inherent part of scientific interest (35, 50). The common term “stem cell” is nowadays used in popular science and increasingly replaced by the more scientific expression of a “multipotent mesenchymal stromal cell (MSC)” because specific stem cell characteristics (51) [long *in vivo* survivability, ability for self-replication and multipotent differentiation into certain tissue types (43)] are insufficiently accurate to prove in therapeutic purposes. However, the term “MSC” is not used uniformly and is not subject to a clear definition. Due to increasing impact of MSCs *via* paracrine effects, the term “medicinal signal cell” has been proposed in recent publications (52, 53).

MSCs can be derived from mesenchymal tissues such as blood, bone marrow and adipose tissue, but do not represent a homogeneous stem cell population (41). In horses, commonly used MSC sources are fat, harvested from subcutaneous adipose tissue at the tail base (lipectomy) (54, 55), bone marrow obtained by puncturing the sternum (56) or venous blood (40, 57). Following tissue harvesting, the process of MSC isolation and cultivation under laboratory conditions requires several weeks to obtain cell numbers usually used for intra-articular applications (41). Besides these autologous cultivated MSCs, commercially available MSC therapeutics are approved by the European Medicines Agency (EMA). Currently, two off the shelf MSC therapeutics are available, one of which uses chondrogenic induced MSCs dissolved in allogeneic plasma (40, 57–59), whereas the other product uses MSCs derived from the umbilical cord (60, 61). These therapeutics contain a defined number of allogeneic MSCs from donor equids. A further alternative to commercially available ready-to-use products is the in-house production of therapeutics from tissue sources like blood, bone marrow or adipose tissue, usually received from the equine patient (autologous) (42). These so-called point-of-care products are readily available through a fast, standardized process of cell separation and MSC enrichment by medical devices (27, 62). Depending on the tissue sources and processing, the final solution contains a variety of different cell types in a mixed population of blood and adipose progenitor cells as well as differentiated cells (41, 42). The proportion of MSC-like cells within the final product is regarded low and not defined (63). With regard to obtain a high number of defined MSCs from the stated tissue sources, MSC isolation and cultivation has to be performed under laboratory conditions (autologous cultivated MSCs) (64, 65). As a result, several millions MSCs are available for application (66). The time between tissue sampling to MSC harvesting calculates several weeks, which must be considered for autologous treatment regimes.

#### 2.1.2. Autologous blood products

Autologous blood products represent a wide range of therapeutics due to the variety of blood processing methods and individual blood components (33, 67). Basically, two groups of blood derived applicable therapeutics can be stated: (1) cell-based and (2) cell-free autologous blood products. For blood processing, commercially available medical devices are provided to equine practitioners.

Cell-based autologous blood products aim to increase the concentration of certain blood cells, mainly platelets, within the applicable therapeutic agent to transmit the regenerative potential of platelet containing growth factors into the joint (68, 69). Depending on the respective blood platelet number and the processing method, the increase in platelet concentration varies widely among products (70). The amount of transmitted growth factors and cytokines depends on the total number of applied platelets, on the injected solution and whether the therapeutic cells are solved in plasma or in a non-blood based injectable solution (71, 72). Platelet rich plasma (PRP) is one of the best-known representatives of this therapeutic group, with a defined 3- to 5-fold increase in platelet concentration in autologous plasma (73, 74). PRP is produced using a double-centrifugation method (41). Alternative processing methods such as single-centrifugation techniques and filtration provide therapeutics with deviating values of platelets and leucocytes from PRP (33, 75). In horses, cellular autologous blood products were commonly used in cases with tendon and ligament injuries (76). However, their use in joint-related diseases is described, and positive outcomes are documented, particularly in combination with MSC-treatments (40, 77). The therapeutic effects have not yet been clarified in detail, since not only growth factors play a pivotal role in tissue regeneration.

Cell-free, serum-based therapeutics represent another group of autologous blood products. After extended coagulation of the patient's blood at 37°C and subsequent centrifugation, the final orthobiologic therapeutic substance provides the full blood cell secretome (26, 78). In addition to the already serum-diluted cytokines, growth factors and proteins, the extended coagulation phase also stimulates *de novo* synthesis of proteins, which enrich the final product to a so far not totally defined extended secretome (79, 80). The mode of action of the acellular autologous blood products is in many aspects not fully defined (81, 82). The often referred increase of anti-inflammatory interleukin-1 receptor antagonist (IL-1Ra) concentration is only partially responsible for the described positive clinical effects (30, 83). The mechanism of action of enriched IL-1Ra as therapeutic agent is to block the receptors and therefore prevent the proinflammatory cytokines interleukin-1β (IL-1β) and tumor necrosis factor alpha (TNF-α) released by the intra-articular inflammatory process from binding (33).

### 2.2. Inclusion criteria

A distinction was made between systematic analysis and meta-analysis. To obtain a general overview, all experimental studies with a follow-up time of more than 6 months were examined in the systematic review. In the meta-analysis, only randomized and controlled trials (RCTs) with a follow-up period of more than 6 months were examined according to the following inclusion and exclusion criteria. To present the results as clearly as possible, the PICO method was used. (a) Population: horses with naturally occurred OA; (b) Interventions: intra-articular therapy by MSCs alone or by MSCs in combination with autologous blood products, or autologous blood products alone; (c) Comparison: degree of lameness before and after intra-articular treatment (comparison of success rate, horses working on competition, horses working at trainings level, lame free horses); (d) Outcome: degree of lameness and adverse effects; (e) Study designs: for the systematic review all experimental studies were included, for the meta-analysis randomized controlled trials were included.

### 2.3. Exclusion criteria

The following studies were excluded: (a) treated animals other than horses and diseases other than OA; (b) use of treatment method other than intra-articular; (c) no clear lameness diagnostics used; (d) not published in English or German; German language was included as this is the authors mother language and articles could be assessed in detail (e) no complete replication of quantitative data of the treated animals (for example individual degree of lameness).

### 2.4. Search strategy

The following research platforms were used (listed according to weighting): PubMed, Google Scholar and CAB direct. Literature searches were carried out using the following keywords: “horse/equine,” “joint/osteoarthritis,” “intra-articular,” “regenerative therapy,” “return/performance.” The search terms could be summarized with the Boolean operators “AND” or “OR” (84). The research was conducted between January 2021 and March 2022. A comprehensive literature search on orthobiologic based joint therapies in horses was undertaken, including all studies published in English and German. This initial investigation summarized 271 findings, of which all studies were examined according to inclusion and exclusion criteria. Subsequently, this initial investigation delivered 86 results. In addition, the reference list of all 86 papers were manually checked for research-relevant studies. To ensure that no meta-analyses relevant to this topic were available, a hit query was performed on PubMed using the two keywords “horse” and “meta-analysis.” The response resulted in 79 meta-analyses. This compares to 18 matches with three real meta-analyses in 2017 (85). These results prove clearly that meta-analysis is becoming more and more relevant in evidence-based medicine. None of these 79 meta-analyses deals in a similar or identical way with the issue investigated in this research. The subsequent table lists the most important studies, sorted by intra-articular administered products, in horses with naturally occurring OA compared to induced OA. In addition, the study duration is indicated >6 months (Table 1).

**Table 1:** Summary of all studies on naturally occurring OA included in the systematic review.

**Mesenchymal stromal cells**									
**References**	**Type of study**	**Number of horses**	**Orthobiologic therapeutic agent and Number of horses per group (n)**	**Treated joint**	**Control group**	**Adverse reactions**	**Time follow up, number of horses completed follow up (n)**	**Outcome: Training level/lame free**	**Outcome: Competition level**
Broeckx et al. (86)	Randomized multicenter double blinded and placebo-controlled study	75	IVP Group (allogenic blood-derived, chondrogenic induced MSCs with equine allogeneic plasma),	Fetlock	Yes	No	1 year *n* = 75	IVP: 19/50 (37%) Placebo: 2/25 (8%)	IVP: 23/50 (47%) Placebo: 0/25 (0%)
Magri et al. (31)	Prospective blinded placebo-controlled study	28	Allogenic umbilical cord-derived MSCs	MCP 16 MTP 6	Yes	Owner detected adverse effects to MSC injection were recorded in 18% of the horses	6 months *n* = 22	8/22 (36%)	5/22 (23%)
Broeckx et al. (77)	Preliminary study	20 (4 × 5)	Group 1: PRP Group 2: allogenic blood-derived, native MSCs Group 3: allogenic blood-derived, native MSCs + PRP Group 4: allogenic blood-derived, chondrogenic induced MSCs + PRP	Fetlock	Yes	No	6 months *n* = 20	Group 1: 0/5 (0%) Group 2: 4/5 (80%) Group 3: 3/5 (60%) Group 4: 4/5 (80%)	
Broeckx et al. (40)	Pilot study	165	Group 1: allogenic blood-derived, native MSCs + PRP, *n* = 49 Group 2: allogenic blood-derived, chondrogenic induced MSCs + PRP, *n* = 116	Coffin (43) Pastern (34) Fetlock (58) Stifle (30)	No	One week after treatment 3 horses had moderate flare reaction	18 weeks Group 1: *n* = 25 Group 2: *n* = 66	Group 1: 11/25 (44%) Group 2: 32/66 (49%)	Group 1: 9/25 (36%) Group 2: 24/66 (36%)
Ferris et al. (18)	Prospective case series	33	Autologous bone marrow-derived MSCs	Stifle	No	3 horses with transient joint flare	24 months *n* = 33	11/33 (33%)	14/33 (42%)
**Autologous blood products**									
**References**	**Type of study**	**Number of horses**	**Treatment and number of horses per group (** ***n*** **)**	**Treated joint**	**Control group**	**Adverse reactions**	**Time follow up, number of horses completed follow up (n)**	**Outcome: Training level/lame free**	**Outcome: Competition level**
Fürst et al. (87)	Prospective randomized controlled trial	30	Group B: GOLDIC^®^ gold-induced autologous-conditioned serum *n* = 16 Group A: betamethasone and hyaluronic acid *n* = 14	Coffin (9) Pastern (1) Fetlock (4) Carpus (8) Tarsus (4) Stifle (3) Shoulder (1)	Yes	Group B: 3/16 mild to moderate (lameness for 24 hours; increased swelling) Group A: 2/14 (joint flare after anesthesia)	> 6 months n group B = 16 n group A = 13	Group B: 3/16 (19%)	Group B: 10/16 (63%) Group A: 6/13 (46%)
Tyrnenopoulou et al. (89)	Placebo controlled study	15	PL	Coffin	Yes	No	1 year	0/10 (0%)	
Bembo et al. (90)	Preliminary clinical study	8	Combination of autologous micro-fat and PRP	Fetlock (7) Carpus (1)	No	No	5–10 months	7/8 (88%)	0/8 (0%)
Bertone et al. (75)	Prospective randomized masked placebo controlled clinical trial	40	APS	MCP (12) MTP (3) Carpus (6) Tarsus (1) Stifle (18)	Yes	No	52 weeks *n* = 38	17/38 (45%)	
Pichereau et al. (91)	Retrospective study	20	PC	Fetlock	No	No	1 year	2/20 (10%)	14/20 (70%)
Jöstingmeier (92)	Prospective study	54	Group 1: Na-Hyaluronat (Hylartil^®^) and Betamethasone (Celestovet^®^) *n* = 27 Group 2: ACS *n* = 27	Coffin	Yes	No	6 months	Group 1: 17/27 (63%) Group 2: 24/27 (89%)	
Carmona et al. (32)	Preliminary pilot clinical study	4	PC	Coffin (1) Fetlock (1) Tarsus (1) Stifle (1)	No	No	1 year	0/4 (0%)	

ACS, Autologous conditioned serum; APS, Autologous protein solution; IRAP, Interleukin 1- Receptor- Antagonist- Protein; IVP, Investigational veterinary product; MCP, Metacarpophalangeal joint; MSC, Mesenchymal stromal cells; MTP, Metatarsophalangeal joint; OA, Osteoarthritis; PC, Autologous platelet concentrate; PL, Platelet lysate; PRP, Platelet rich plasma.

### 2.5. Data extraction

To meet the aim of the topic, only *in vivo* studies were analyzed. For the systematic review, all experimental studies were included regardless their level of evidence or design, with and without a control group. The control group was defined as another horse, another leg (contralateral limb) or another treatment method. The following data were examined and listed according to the following aspects: author, year of publication, type of study (RCTs/ No-RCTs), sample size, treatment protocol, treated joint/joints, placebo-controlled, adverse reactions, follow-up time, lameness evaluation (horses working at trainings level/lame free horses/horses working on competition level/success rate).

For the intra-articular treatment regimen with orthobiologic therapeutics, there were no specifications regarding diagnostic methods, treatment frequency, dosage, and preparation of the appropriate therapeutics (intra-articular therapeutics with MSC and/or autologous blood products are allowed). In addition, studies with any joint with naturally occurring OA were included in the systematic review; there were no specifications on a specific localization. Finally, all studies were evaluated based on the lameness examination and classified into either a positive or a negative outcome. The positive outcomes were divided into two groups: horses working at training level and horses returning to competition. Horses with a negative outcome did not respond to treatment or had a relapse during the observation period. For the meta-analysis, the two positive outcome groups (horses working at training level/lame free and horses returning to competition) were combined due to a lack of study numbers.

### 2.6. Quality assessment

Each study in the systematic review was examined for the following 7 bias characteristics: random sequence generation (selection bias), allocation concealment (selection bias), blinding of participants and personnel (performance bias), blinding of outcome assessment (detection bias), incomplete outcome data (attrition bias), selective reporting (reporting bias), other source of bias. Regarding each aspect, the studies were classified as high risk, low risk or unclear risk according to the PRISMA guidelines (93). For a better visualization, a traffic light table with “high risk” in red, “low risk” in green, and “unclear risk” in yellow was created. The classification into the category “unclear risk” occurs when relevant details for the classification into bias are not sufficiently substantiated in the respective study (94).

### 2.7. Statistical analysis

Using the PRISMA guidelines, 13 studies met the inclusion criteria for the systematic review (93). To compare dichotomous outcomes *via* meta-analysis, an odds ratio (OR) with 95% confidence interval (CI) was calculated using the R program (95). For data evaluation a random-effects model was used describing the overall outcome. Each study with its estimated effect size and corresponding confidence interval is graphically represented in the forest plot. Furthermore, the forest plot illustrates the extent to which the result from the individual study varies (96, 97). This variability is referred to as heterogeneity and is assessed by I^2^ in the following meta-analysis. Heterogeneity was determined to be significant at I^2^ > 50% or *p* < 0.1. A result was considered significant with *p* < 0.05.

### 2.8. Meta-analysis

In the meta-analysis, the results of lameness evaluation at different time periods of the studies were presented in the individual sections. In the short-term follow-up periods, one additional placebo-controlled and randomized trial was examined for better comparability (57). These will be discussed separately. All long-term studies are listed in the last row of the forest plot.

## 3. Results

### 3.1. Risk of bias

With all instruments that measure the risk of bias in clinical trials, it must be considered that they do not present an exact measurement method. Instead, it is an estimation in which the result always contains a subjective component. The purpose is to compare similar and homogenous treatment groups affected only by random variabilities (75).

**Figure 1:**
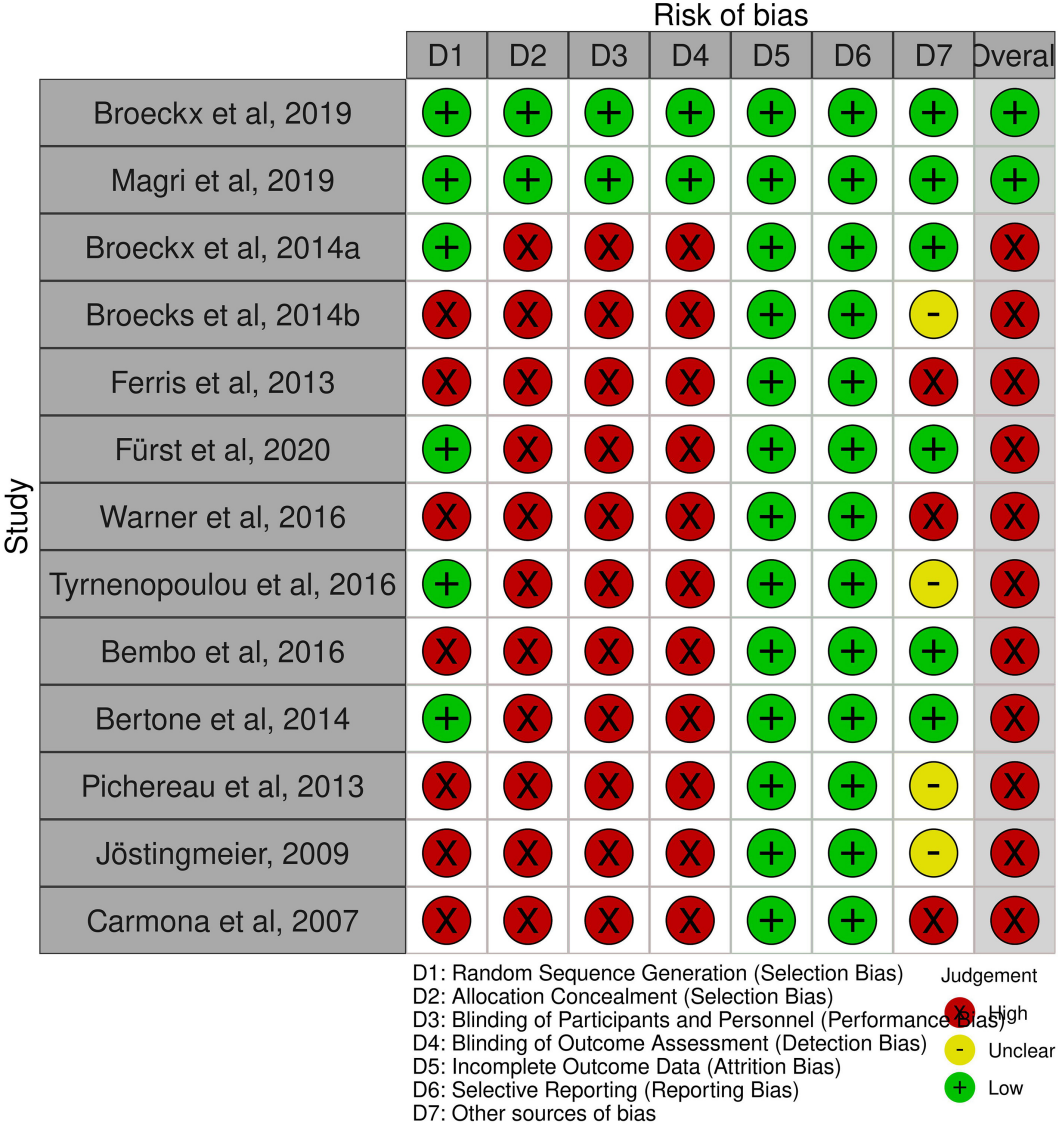
Summary of risk of bias (98).

All studies in the systemic review were assessed against the listed seven criteria and classified as high, low, or unclear risk (Table 2). The traffic light system (Figure 1) was used to illustrate the overall risk achieved by each study. Six studies avoided selection bias by randomly assigning participants (31, 75, 77, 86, 87, 89). Secrecy of the randomization scheme and blinding of veterinarians and patient owners was met by only two studies, both demonstrate a low risk of bias (31, 86). Blinding of treatment was achieved by generating two groups of examining and dispensing veterinarians at both study sites and owner's absence at administering the agent (86). For comparison, in the other study, the syringe was blinded so that owners and veterinarians did not know which treatment regime was selected. Blinding was maintained throughout the entire duration of the study (31).

**Table 2:** Studies included in the systematic review demonstrating different source of bias.

**References**	**Random sequence generation (selection bias)**	**Allocation concealment (selection bias)**	**Blinding of participants and personnel (performance bias)**	**Blinding of outcome assessment (detection bias)**	**Incomplete outcome data (attrition bias)**	**Selective reporting (reporting bias)**	**Other source of bias**
Broeckx et al. (86)	Low	Low	Low	Low	Low	Low	Low
Magri et al. (31)	Low	Low	Low	Low	Low	Low	Low
Broeckx et al. (77)	Low	High	High	High	Low	Low	Low
Broeckx et al. (40)	High	High	High	High	Low	Low	Unclear
Ferris et al. (18)	High	High	High	High	Low	Low	High
Fürst et al. (87)	Low	High	High	High	Low	Low	Low
Warner et al. (88)	High	High	High	High	Low	Low	High
Tyrnenopoulou et al. (89)	Low	High	High	High	Low	Low	Unclear
Bembo et al. (90)	High	High	High	High	Low	Low	Low
Bertone et al. (75)	Low	High	High	High	Low	Low	Low
Pichereau et al. (91)	High	High	High	High	Low	Low	Unclear
Jöstingmeier (92)	High	High	High	High	Low	Low	Unclear
Carmona et al. (32)	High	High	High	High	Low	Low	High

**Figure 2:**
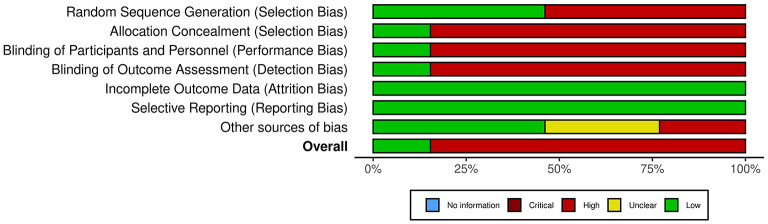
Risk of bias (98).

All studies in the systematic review reported study discontinuations and missing outcome data. Therefore, almost all studies were considered to have a low risk of incomplete results and selective reporting. In addition, most studies used an owner questionnaire for long-term follow-up. In summary, eleven studies are at a high risk of bias (18, 32, 40, 75, 77, 87–92), due to the lack of blinding. The risk of bias graph shows the authors' assessment of each item in percentage (Figure 2). Overall, <25% of the studies included in the systematic review were found to be at a low risk of bias.

### 3.2. Systematic review

**Figure 3:**
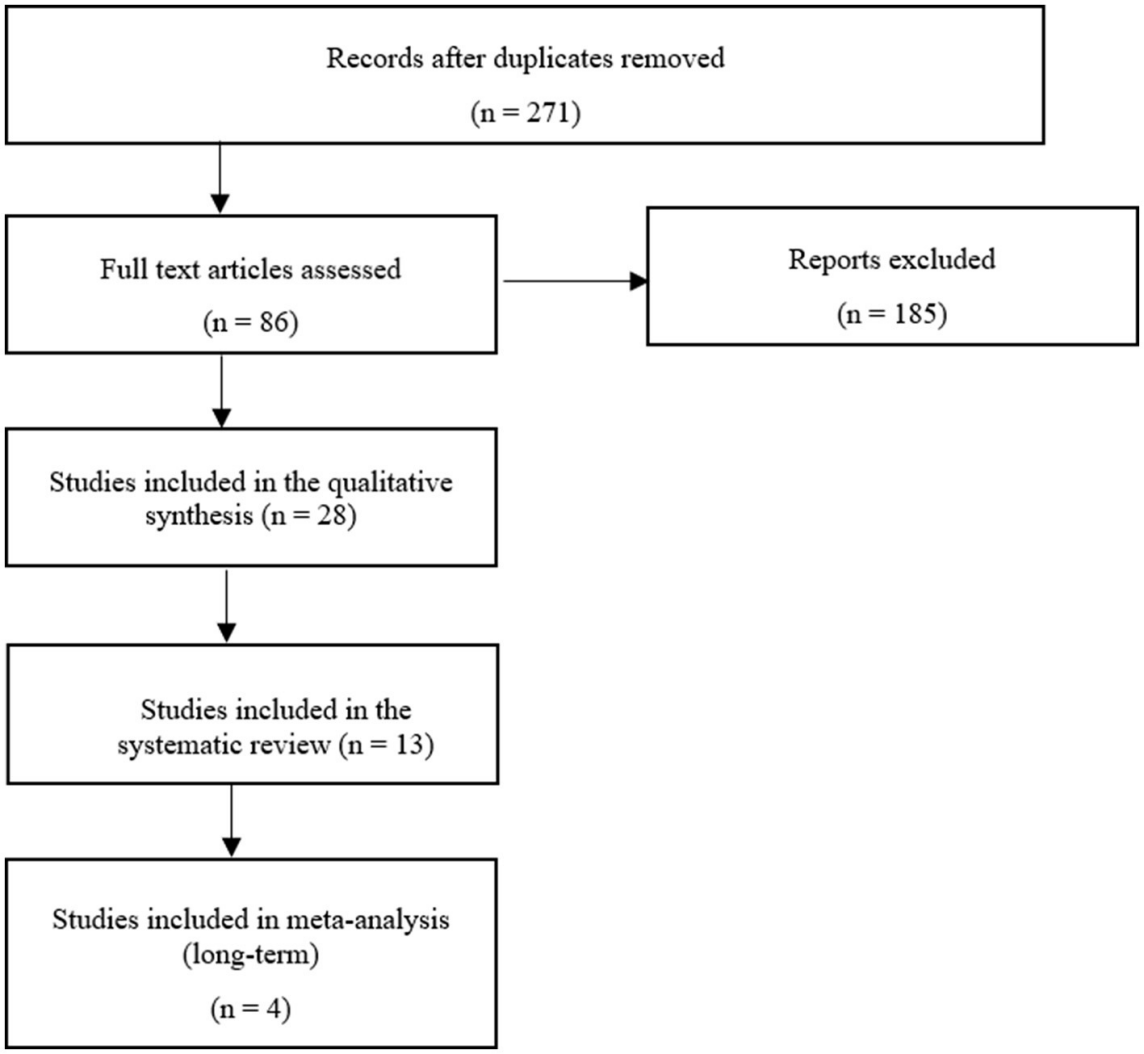
Flow chart showing the methods used for the systemic search (93).

The flowchart (Figure 3) shows the detailed systematic analysis after initial electronic and manual research, with a total of 271 studies. This resulted in 28 studies being assessed for the qualitative synthesis after an initial review. These studies were further assigned to the defined orthobiologic therapies when treatment of equine OA was the scientific focus (Table 3). Following this, the biologic cell source of the selected 28 studies was assessed and listed (Table 4). This states, that based on their sources of orthobiologic therapeutics, 4 studies were included using blood-derived MSCs either non-induced (native) or chondrogenic-induced (40, 57, 77, 86), 5 studies focused on bone marrow-derived MSCs (18, 29, 99–101) from which 2 studies also included MSCs derived from adipose tissue for comparison (100, 101). Five studies included adipose tissue-derived MSCs only (27, 55, 100–102). The effect of umbilical cord-derived MSCs were studied in 2 publications (31, 61). Eight studies describe the use of cell-based autologous blood products as orthobiologic therapeutics (19, 32, 75, 89–91, 103, 104) and 6 studies a cell-free final therapeutic product (30, 81, 87, 88, 92, 105). The following results were obtained: 8 studies used MSCs as a therapeutic agent for naturally occurring OA (18, 31, 40, 55, 61, 77, 86, 102); 6 studies examined the effect of MSCs after inducing OA (27, 29, 57, 99–101); 14 studies treated with autologous blood products, with 1 study inducing OA (30) while the remaining studies examined naturally occurring OA (19, 32, 75, 81, 87–92, 103–105). Of the 14 MSC-related studies, 12 were placebo controlled (27, 29, 31, 55, 57, 61, 77, 86, 99–102) and 7 studies had an outcome with patient follow-up at least 6 months after treatment initiation (18, 27, 29, 31, 77, 86, 99). Comparatively, of the 14 groups treated with autologous blood products, 7 were placebo controlled (19, 30, 75, 87, 89, 92, 103) and 8 studies had a long-term follow-up (32, 75, 87–92). After screening the studies with the specified inclusion and exclusion criteria, 13 studies were examined for systematic analysis, and listed in Table 1 (18, 31, 32, 40, 75, 77, 86–92).

**Table 3:** Numbers of studies from qualitative synthesis including naturally occurring OA compared to induced OA, trials with a placebo group, and studies with a follow-up time over 6 months.

**Product**	**Naturally occurring OA**	**Induced OA**	**Placebo**	**Outcome > 6 months**
Mesenchymal stromal cells (MSC)	8	6	12	7
Autologous blood products	13	1	7	8
Total number qualitative synthesis	28		

OA, Osteoarthritis.

**Table 4:** Numbers of studies from qualitative synthesis demonstrating the source and composition of orthobiologic therapeutic agents.

**Product**	**Blood-derived**	**Bone marrow-derived**	**Umbilical cord-derived**	**Adipose tissue-derived**	**Blood cell-based**	**Blood cell-free**
Mesenchymal stromal cells (MSCs)	4 (40, 57, 77, 86)	5 (18, 29, 99–101)	2 (31, 61)	5 (27, 55, 100–102) +2 (100, 101)		
Autologous blood products					8 (19, 32, 75, 89–91, 103, 104)	6 (30, 81, 87, 88, 92, 105)

MSCs, Mesenchymal stromal cells.

Two studies (100, 101) are double reported due to the use of both, bone marrow-derived and adipose tissue-derived MSCs.

Table 5 lists all 15 studies from the quantitative synthesis that were not included in the systematic review due to the lack of information on the individual degree of lameness. Therefore, an average value was given for the whole group. Other reasons for exclusion were short observation periods, induced OA, or an overall too short observation time.

**Table 5:** Studies from literature review that are not included in the systematic review because of incomplete data referring to the set inclusion criteria.

**References**	**Title**	**Exclusion criteria**
McIlwraith et al. (29)	Evaluation of intra-articular mesenchymal stem cells to augment healing of microfractured chondral defects	Defects arthroscopically created; second-look arthroscopy at 6 months; lameness effects were not reported for each horse individually
Mariñas-Pardo et al. (55)	Allogeneic adipose-derived mesenchymal stem cells (Horse Allo 20) for the treatment of osteoarthritis associated lameness in horses: characterization, safety and efficacy of intraarticular treatment	Follow up 90 days; lameness effects were not reported for each horse individually
Frisbie et al. (101)	Evaluation of adipose-derived stromal vascular fraction or bone marrow-derived mesenchymal stem cells for treatment of osteoarthritis	Osteoarthritis was induced arthroscopically; follow up 70 days; lameness effects were not reported for each horse individually
Broeckx et al. (57)	The use of equine chondrogenic-induced mesenchymal stem cells as a treatment for osteoarthritis: A randomized, double-blinded, placebo-controlled proof-of-concept study	Osteoarthritis was induced using an osteochondral fragment-groove model; follow up to week 11 *Study was included in the meta-analysis as short-term study due to the randomized, double-blinded, placebo-controlled study design*
Frisbie et al. (100)	Evaluation of bone marrow derived stem cells and adipose derived stromal vascular fraction for treatment of osteoarthritis using an equine experimental model	Osteoarthritis was induced arthroscopically; follow up 8 weeks; lameness effects were not reported for each horse individually
Pradera Muñoz (61)	Efficacy and safety study of allogeneic Equine Umbilical Cord derived Mesenchymal-Stem Cells (EUC-MSCs) for the treatment of clinical symptomatology associated with mild to moderate degenerative joint disease (osteoarthritis) in horses under field conditions	Follow up 63 days; many data were lost during the 2 years follow up; *Study included in the meta-analysis as short-term study due controlled, blinded, randomized study design*
Mirza et al. (104)	Gait Changes Vary among Horses with Naturally Occurring Osteoarthritis Following Intra-articular Administration of Autologous Platelet-Rich Plasma	Horses did not respond to intra-articular anesthesia with a consistent pattern of gait changes as expected from responses; lameness effects were not reported for each horse individually
Frisbie et al. (30)	Clinical, biochemical, and histologic effects of intra-articular administration of autologous conditioned serum in horses with experimentally induced osteoarthritis	Osteoarthritis was experimentally induced; follow up 70 days; lameness effects were not reported for each horse individually
Weinberger (105)	Klinische Erfahrungen mit der Anwendung von ACS/ORTHOKIN/IRAP beim Pferd Clinical experience with the application of ACS/ORTHOKINE/IRAP in horses	Not placebo controlled; follow up time 12 weeks
Nicpoń et al. (102)	Therapeutic effect of adipose-derived mesenchymal stem cell injection in horses suffering from bone spavin	Insufficiently detailed case numbers about working at trainings level or success rate
Abellant et al. (103)	Intraarticular platelet rich plasma (PRP) therapy evaluation in 42 sport horses with OA	Publication in IVIS only; not published in a peer reviewed journal
Smit et al. (19)	Clinical findings, synovial fluid cytology and growth factor concentrations after intra-articular use of a platelet-rich product in horses with osteoarthritis	Follow up 56 days; no lameness evaluation because due to unforeseen external factors
Barrachina et al. (99)	Assessment of effectiveness and safety of repeat administration of proinflammatory primed allogeneic mesenchymal stem cells in an equine model of chemically induced osteoarthritis	Follow up 6 months; lameness effects were not reported for each horse individually
Marques-Smith et al. (81)	Is clinical effect of autologous conditioned serum in spontaneously occurring equine articular lameness related to ACS cytokine profile?	Follow up mean 48 days; lack of control
Yamada et al. (27)	Mesenchymal stem cells enhances chondral defects healing in horses	Experimentally induced OA; lameness effects were not reported for each horse individually

The age, sex, breed, and disposition of the horses selected for the investigations varied among the studies. Most of the trials in the systematic review examined the effect of orthobiologic therapeutics for the coffin or fetlock joint (Table 1). Five of the 13 studies treated naturally occurring OA with MSCs (18, 31, 40, 77, 86), and 1 study subdivided the treatment groups into f4 subgroups (PRP; native MSCs, native MSCs with PRP; chondrogenic-induced MSCs with PRP) (77). This study used allogenic peripheral blood as MSC source and labeled isolated, non-induced MSCs as “native.” Due to lack of placebo-controlled studies, the PRP-subgroup was compared with the MSC-subgroups of different sources in combination with PRP in the following meta-analysis. The remaining studies treated horses with non-induced (“native”) MSCs, chondrogenic-induced MSCs, umbilical cord-derived MSCs and bone marrow-derived MSCs. None of the 5 reviewed studies using adipose-derived MSCs as orthobiologic agent met the inclusion criteria for meta-analysis (27, 55, 100–102). In general, all MSC-studies demonstrated a heterogenous group regarding manufacturing and processing methods of the particular cell source. Considering possible side effects, 3 studies observed a mild to moderate inflammatory response after intra-articular treatment with MSCs (18, 31, 40). At the final examination, all patients felt well. Therefore, no general side effects were concluded.

Eight of the 13 studies examined treatment outcomes with autologous blood products. Of these, 3 studies used autologous conditioned serum (ACS) products (87, 88, 92). The remaining studies used cellular autologous blood products with a high platelet-rich content (32, 75, 89–91). Mild side effects such as self-limiting local swelling and lameness were noted in 1 ACS study (87).

Concerning the post-treatment, every study designed a particular rehabilitation program. All horses received a 1- (86, 89) to 8-week (31) hand-walking program at the end of treatment, followed by individual retraining. Most studies graded the severity of lameness according to the AAEP (American Association of Equine Practitioners) scoring system (18, 32, 75, 77, 86–90).

Although several placebo-controlled studies were included, most of them lack long-term follow-up or control was not maintained throughout the entire duration of the study. For example, in one RCT, horse owners in the placebo group were offered treatment with autologous protein solution (APS) 14 days after the placebo treatment. The randomized controlled study was well-structured, but the observation time of the control group was too short to be included in our meta-analysis. The APS group improved significantly after the treatment compared with baseline or control group scores (75). In total, 5 studies were placebo controlled over the entire observation period, 4 of which were randomized (77, 86, 87, 89). These studies have also been included in the meta-analysis.

**Figure 4:**
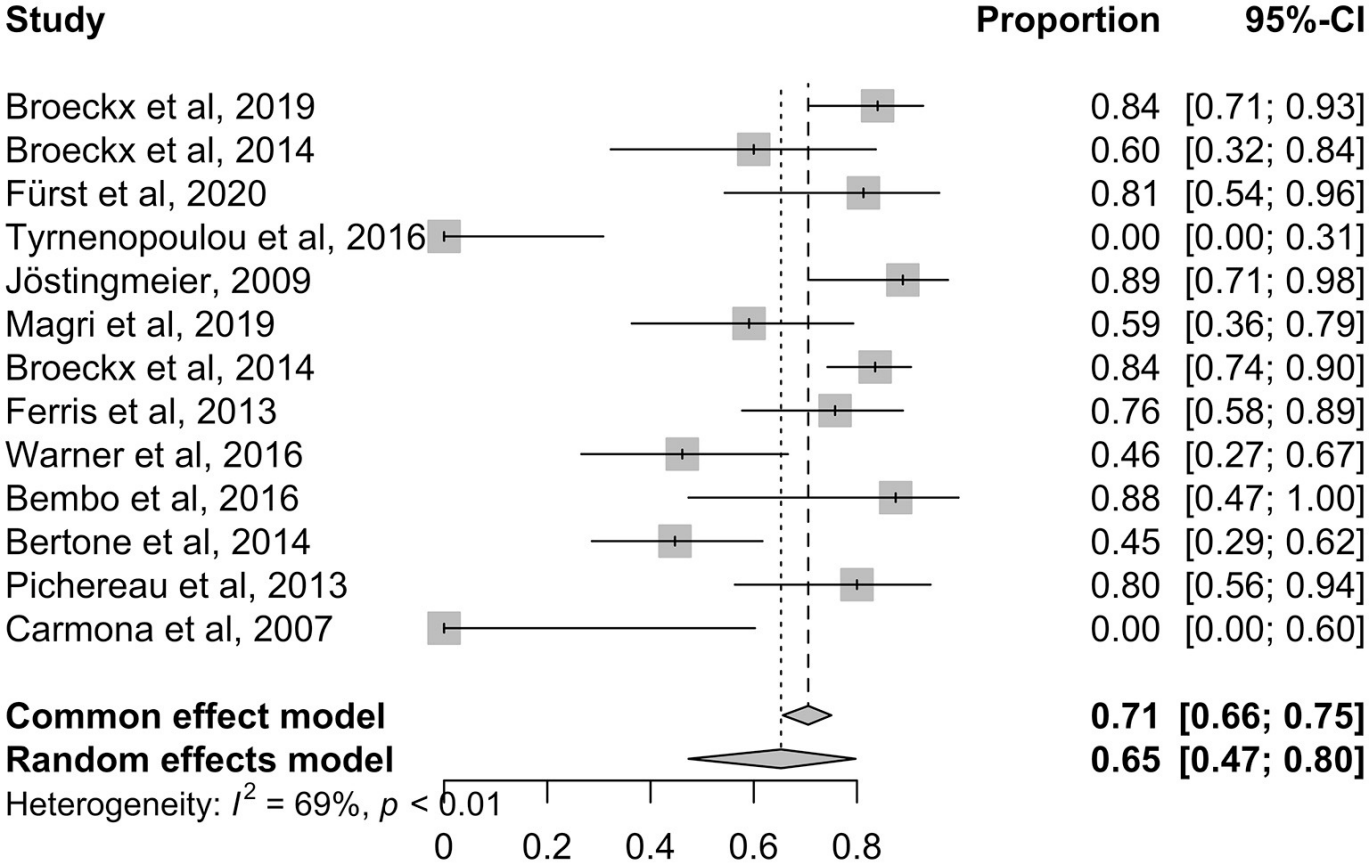
Shows the ratios of the individual studies in the systematic analysis graphically with outliers.

**Figure 5:**
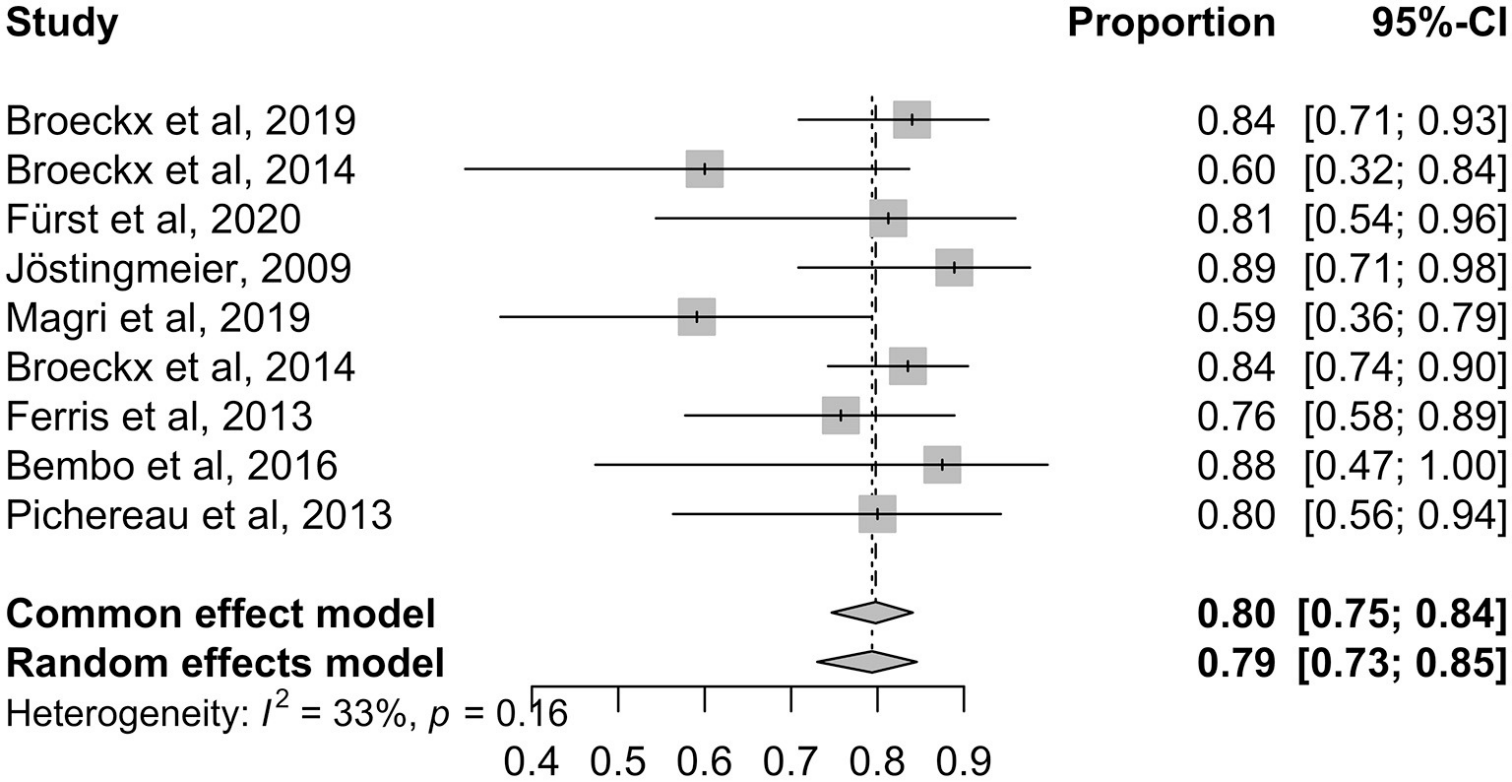
Shows the ratios of the individual studies in the systematic analysis graphically without outliers.

In summary, an average of 65% improvement in lameness grade was achieved after the treatment with intra-articular applied orthobiologic therapeutics, regardless of which therapeutic agent was used (Figure 4). Eleven studies showed a general positive effect after treatment, with horses working at trainings level or horses returning to competition (Table 1). Two outliers could be detected, that showed initial improvement in the first 7–8 months after treatment but then returned to their initial degree of lameness (32, 89). In both studies, the majority of horses responded positively at the beginning and maintained their high level of performance over a period of at least 6 months. Furthermore, horses showed no adverse reactions. This outcome suggests that platelet lysate (PL) and autologous platelet concentrate (PC) can be an efficient short-term therapy for horses suffering from OA (32, 89) (Figure 5). Looking at the average proportions without outliers, 80% of the horses involved in the studies showed lameness reduction after treatment with orthobiologic therapies (Figure 5).

As previously stated, in 1 study treatment groups were divided into 4 subgroups, and interestingly, the group treated with chondrogenic induced MSCs had the most successful result, with 80% lameness-free horses and horses working at trainings level (77). Another pilot study compared non-induced (native) MSCs with chondrogenic induced MSCs, both in combination with PRP. This resulted in a higher average score for the beneficial effects using chondrogenic induced MSCs. However, the result was statistically non-significant (40).

Promising results with MSCs and ACS were justified over a 24-months follow-up period (18, 88). After intra-articular administration of MSCs postoperatively after arthroscopy of the stifle, 42% of horses returned to their previous level of work, and 33% returned to work after a mean follow-up period of 24 months (18). In a retrospective study from Warner et al. 31% of the horses returned to their previous level of work and 15% performed at exercise level after a period of at least 2 years following the ACS treatment of the coffin joint (88). Both studies were not blinded and without a control group, which significantly limits their validity. However, both studies provide indication of a long-term effect of MSC and ACS treatment in OA.

### 3.3. Meta-analysis

Four RCTs (77, 86, 87, 89) out of the 13 trials were included in the long-term meta-analysis with a follow-up time >6 months. The control groups were treated with saline (86, 89), other orthobiologic therapeutic agents (PRP) (77) or corticosteroids and hyaluronic acid (87). All studies included horses of different breed, sex, age, and level of performance. Moreover, the diagnosed and treated OA occurred in different joints, ranging from low to high motion joints (Table 1). Due to the scarcity of studies, no restrictions were made.

**Figure 6:**
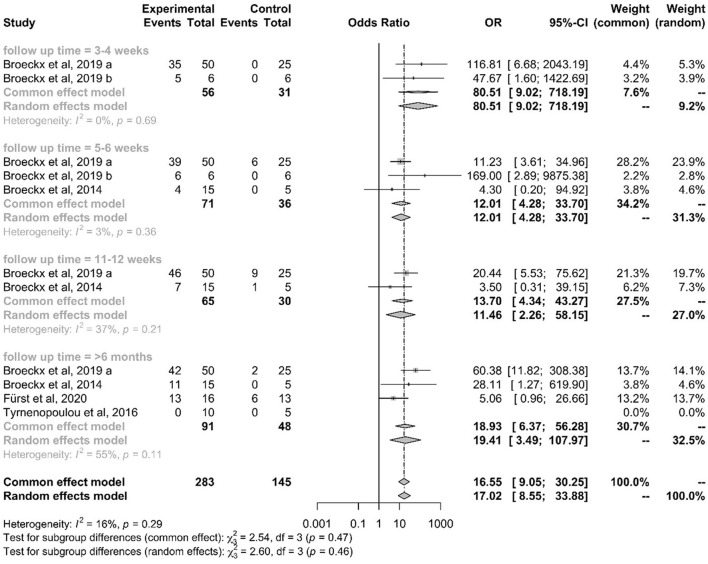
Forest plot showing results of selected studies using a meta-analysis to compare lameness reduction of experimental and control. The common effect model and the random effects model are shown. Depending on heterogeneity (I^2^ > 50%) the random effects model was used for studies with long-term follow-up. The greater the squares, the more participants included the study. The size of the squares is proportional to the weight of the study. The whiskers correspond to the 95% confidence interval (Cl).

Figure 6 demonstrates a forest plot with outcomes at different time points. The focus was the set inclusion criteria of a follow-up period >6 months. Three studies (77, 86, 87) showed a positive impact of orthobiologic therapeutics compared to their control groups.

One study reported a regression to its initial lameness level after an observation period of 1 year (89). In this study, no side effects were noted in the first 6 months after treatment and 9 out of 10 horses treated with PL returned to their normal activity. Lameness recurred from the 7th month, and all horses relapsed to their initial degree of lameness at the end of the study period. This study illustrates the correlation between duration of follow-up and recurrence of lameness. Within 6 months, horses returned to their previous level of performance. However, all horses relapsed to their initial degree of lameness, therefore only a temporary positive effect could be observed.

The forest plot illustrates the common effect model and the random effects model and whether heterogeneity could be stated as significant. Data demonstrating I^2^ > 50% were assigned to the random effects model. As demonstrated in the last row of the plot, all long-term follow-up studies showed moderate heterogeneity with I^2^ = 55% and *p* = 0.11 (Figure 6). A random effects model was used due to the assumption of moderate differences among study design and implementation in the clinical studies.

An odds ratio (OR) of 1 indicates no difference between the treatment and control group, whereas an OR > 1 indicates that lameness is more likely to be reduced in the experimental group. All studies with an OR values higher than 1 favor the experimental group (Figure 6: OR 17.02; 95% CI: 8.5474 to 33.8849 *p* < 0.0001). None of the studies crossed the line into ineffectiveness, suggesting that the treatment effect was estimated to be similar across studies.

The diamond square represents the average of all individual studies. If the limit of ineffectiveness is not exceeded, a significant difference in lameness reduction between the experimental and control groups is stated. It can be summarized, that the included orthobiologic therapeutics are safe and showed significant improvement in lameness reduction compared to their control groups. Three studies (77, 86, 87) showed a long and constant improvement over 6 months.

One short-term RCT was included in the forest plot for comparative reasons (57). We assessed at what time point the trial showed significance for treatment with an orthobiologic therapy and how effective the short-term trial was. Treatment success with chondrogenic induced MSCs in an induced OA model was demonstrated to be a time dependent factor, with decreasing lameness levels from 2 weeks after treatment throughout the observation period of 11 weeks (57).

In summary, the use of intra-articular administered orthobiologic therapeutic agents show an incidence of lameness reduction by 73% compared to the control in the long-term follow-up, whereas in the control group lameness was reduced by 17% (77, 86, 87, 89). According to the included studies, horses with naturally occurring OA demonstrated a significantly reduced degree of lameness after intra-articular treatment with orthobiologic therapeutics compared with the control in the long-term follow-up.

### 3.4. Publication bias

Publication bias occurs when the probability of a study being published depends significantly on its outcome. This means that it is more likely, that a study will be published if the results are consistent with the hypothesis or if the study results are significant (106).

The occurrence of publication bias can be tested by creating a funnel plot. Ideally, the individual data points form a symmetrical, inverted funnel. On the x-axis, the treatment effect is plotted against the study size on the y-axis. The largest studies are located at the top of the graph and plotted near the average. The smaller studies are distributed on both sides of the average and lie close to the x-axis.

**Figure 7:**
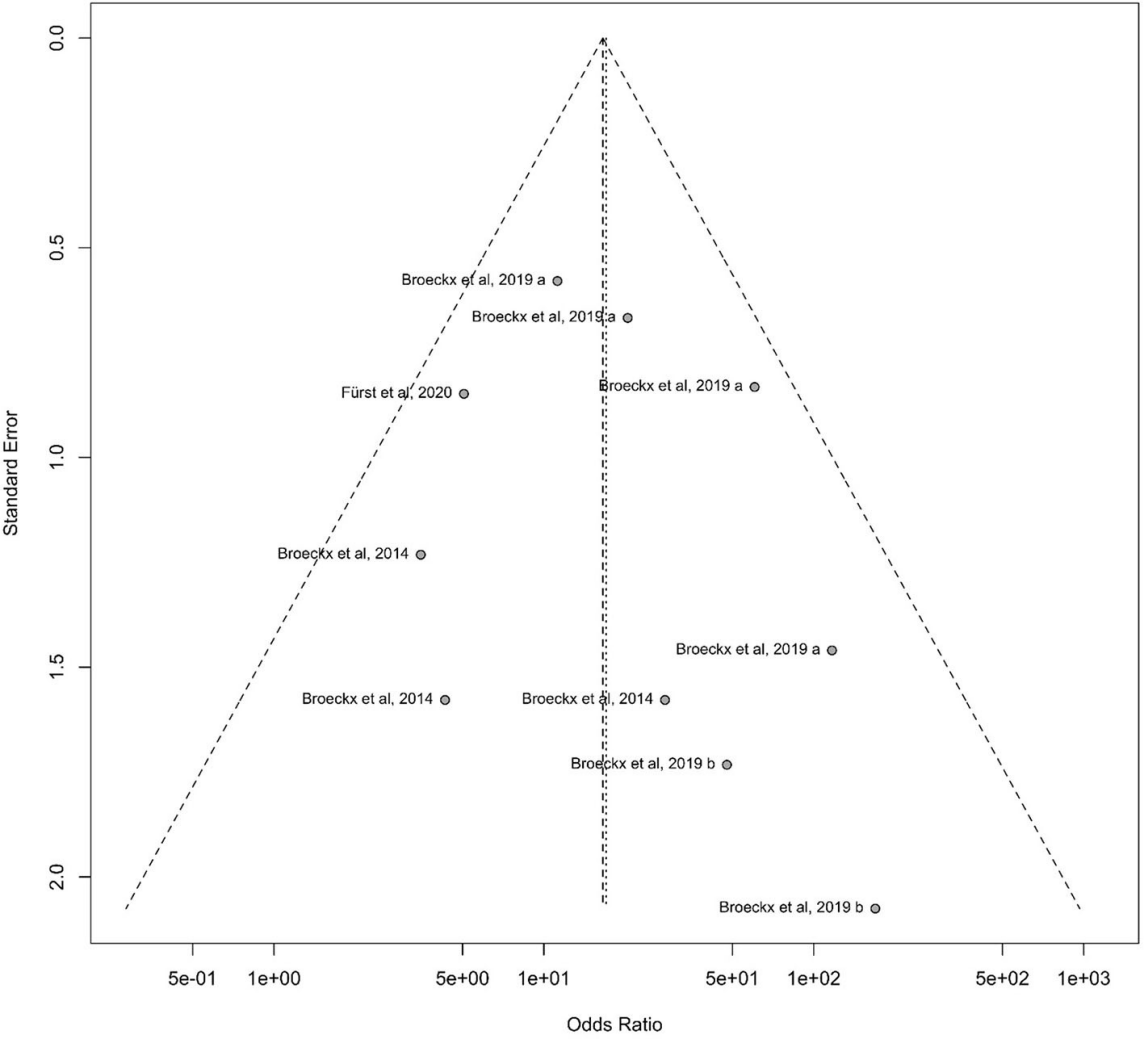
Funnel diagram demonstrating the standard error to the odds ratio according to the study design of participating horses. On the x-axis, the treatment effect is plotted against the study size on the y-axis. Largest studies are located at the top of the graph and plotted near the average. Smaller studies will spread on both sides of the average and lie close to the x-axis.

The funnel plot showed almost the desired symmetrical shape, with the studies close to the midline. It is important to note, that studies that conducted lameness examinations at different time points are considered as individual studies. For instance, a study by Broeckx et al. (57) is plotted 4 times in the funnel plot, at each study time point. The hypothesis that studies with a smaller number of participants are more likely to be in the bottom range is correct. Overall, the publication bias can be classified as low (Figure 7).

## 4. Discussion

OA is a leading cause of pain, disability and economic impact on the health system worldwide (3, 107). The demand for regenerative medicine to treat OA is steadily increasing in human and veterinary medicine. Therefore, it is important to obtain an up-to-date state of knowledge and to compare previous studies using meta-analysis (108, 109). There are two main reasons why the equine model is a suitable model for human medicine. First, horses spontaneously develop chondral defects and age-/trauma-induced OA that are very similar to humans (15). Second, there are numerous *in vitro* and *in vivo* studies, some even with experimentally induced OA, in which the therapeutic index of orthobiologic therapeutics can be assessed (109).

To our knowledge, this is the first meta-analysis comparing orthobiologic therapies with its control group in long-term *in vivo* studies for the treatment of OA. Overall, many topic-related articles were recorded, but of the 86 articles fully screened, only 13 (15%) were useful for the systematic review after passing the inclusion and exclusion criteria. Finally, only 4 (5%) of these studies could meet the criteria for the meta-analysis (Figure 3). This shows that although there is a great research interest in this topic area only a few studies examine long-term success compared with a control group. The result of this meta-analysis showed comparable studies with a moderate heterogeneity, which overall demonstrate a positive result in terms of orthobiologic therapy (Figure 6). By demonstrating the therapeutic efficiency of the mentioned therapies in the long-term in clinical cases of OA, the application of such therapeutics in equine veterinary practice is justifiable.

Major limitations were, that the number of comparable studies that met the inclusion criteria were low. Most studies suitable for systematic review lacked a control group. Another shortcoming was the absence of a uniform treatment pattern in the controlled trials. All controlled studies treated with different placebos [saline (86, 89), other potentially regenerative agents (77), cortisone and hyaluronic acid (87)]. Compared to other meta-analysis and systematic reviews, the lack of an adequate placebo group was also the main point of criticism (70, 110). From an animal welfare perspective, it is unethical to not treat animals suffering from joint-related pain. Moreover, it is difficult to find a homogenous control group, in which all horses are treated with the same agent. However, it is almost impossible to convince horse owners to participate in a long-term study without them knowing whether they will be receiving a placebo or a treatment. Especially, since there is a real chance that their horse will miss out on a potential therapy. In general, all privately owned horse owners wanted to be assured that everything was being done to get the horse well and back to work.

The lack of blinded study designs in RCTs is noticeable. Overall, only 2 long and 2 short-term studies were fully blinded (31, 57, 61, 86). Reasons for this include the high effort of blinding all medical staff and owners. In addition, it is often difficult to obtain permission from horse owners for placebo-controlled and blinded study participation for the entire study duration. The absence of blinding is often associated with excessive reasoning, especially when assessing subjective outcomes (111, 112). The lack of blinding is the main reason for the high risk of bias.

Another serious point of criticism is the difference of the joint localization. Due to the lack of studies, no restriction was made here, and all long-term studies could participate, regardless of the joint in which the OA occurred. The emphasis was placed on lameness reduction in a long-term follow-up. Of course, from a medical point of view, there is criticism on the comparability of the individual joints. No distinction was made between chronic or acute OA, mild or advanced OA. The absence of a homogenous concept shows the need for further studies. To avoid heterogenicity, a meta-analysis with naturally occurring chronic OA in the same joints would be useful.

The systematic analysis showed a positive result of 80% in all studies, except for the two outliers. In other words, over 80% of the horses treated with orthobiologic therapies showed a reduction in their degree of lameness. Lameness evaluation was uniformly investigated in 9 studies using the AAEP score (18, 32, 75, 77, 86–90); the other studies used their own clinical scores. In most studies, the endpoint survey was conducted using an owner survey. Although the owners' assessment is subjective, comparability can be established because health status and degree of lameness are collected in relation to the performance level before and after treatment.

Two short-term studies were double-blind, randomized, and placebo-controlled with a low potential for bias. This showed that a very safe study design is possible in studies with a shorter control period, as blinding can be maintained (57, 61). In summary, significant lameness improvement with orthobiologic therapy was observed in both groups from the 2^nd^ (57) and 5^th^ (61) week after treatment. In these models, accurate experimental design and maintenance of blinding is facilitated. However, most animal models are limited to a period of 8 to 12 weeks (Table 5). In addition, many studies reported only an average or mean values for lameness evaluation. Individual results are usually missing here (30, 57, 100, 101). Due to a missing randomization scheme and blinding, many studies show a high potential for bias.

Overall, moderate heterogeneity among the studies in the meta-analysis has been described. All product- and treatment-specific factors mentioned above have an unknown impact on treatment success. The aim of this study is to draw attention to the importance of a correct study design. The results indicate a significant improvement with orthobiologic therapies compared to their control for at least several months. However, due to the paucity of studies with long-term and placebo-controlled follow-up, no concrete statement can be made regarding effectiveness of specific orthobiologics, exemplary the preference of MSCs to autologous blood products and vice versa. However, equine practitioners can rely on a safe and effective treatment option when using orthobiologics but thereof no recommendation regarding specific products can be derived. In the future, more randomized, controlled, blinded studies and long-term studies are needed to make further informed conclusions. It is crucial to determine the exact composition and effect of all orthobiologic therapeutics in further studies to develop effective and standardized treatment protocols.

## 5. Conclusion

Apart from the limited and sometimes controversial findings, the systematic review and meta-analysis showed an overall support toward the orthobiologic therapeutic application. After treatment with orthobiologics, a beneficial effect on OA was demonstrated without significant adverse effects. Satisfactory effects were examined over a period of 6–12 months, with a high success rate. Limitations lie within the lack of homogeneous standardization protocols and outcome measurements. Future studies should focus on standardized study designs regarding patient details, treated joints and type of orthobiologic substances in RCTs to allow comparable conclusions about the long-term effect of intra-articular administered orthobiologic therapeutics.

## Author contributions

AM, AT, YZ, and SR constructed the manuscript. AT, SR, and WB edited the manuscript. All authors contributed to the article and approved the submitted version.
